# Utilization of Hematite Particles for Economical Removal of *o*-xylene in a High-Temperature Gas-Solid Reactor

**DOI:** 10.3390/molecules27051509

**Published:** 2022-02-23

**Authors:** Xiaolong Ma, Dandan Zhao, Jinjin Qian, Zichuan Ma, Jiansheng Cui

**Affiliations:** 1 School of Environmental Science and Engineering, Hebei University of Science and Technology, Shijiazhuang 050018, China; maxiaolong2410@hebust.edu.cn; 2 Hebei Key Laboratory of Inorganic Nano-Materials, College of Chemistry and Material Sciences, Hebei Normal University, Shijiazhuang 050024, China; zhaoddv@163.com (D.Z.); qianjinjin0209@163.com (J.Q.)

**Keywords:** *o*-xylene, hematite, reduction, removal, metallic iron

## Abstract

To establish a novel approach for VOCs resource utilization, coupled *o*-xylene oxidation and hematite reduction was investigated in this study in a high-temperature gas-solid reactor in the temperature range 300–700 °C. As the *o*-xylene-containing inert gas (N_2_) stream traveled through the hematite particle bed, its reaction behavior was determined in programmed heating and constant temperature modes. Consequently, the effect of bed temperature, flow rate and *o*-xylene inlet concentration on both *o*-xylene removal performance and degree of hematite reduction was studied. The raw hematite and solid products were analyzed by TGA, XRF, XRD and SEM-EDS. The results showed that a temperature above 300 °C was required to completely eliminate *o*-xylene by hematite, and both *o*-xylene removal capacity and degree of hematite reduction at 5% breakthrough points enhanced on increasing the temperature and decreasing the flow rate. The increment in temperature from 300 °C to 700 °C led to a gradual reduction of Fe_2_O_3_ to Fe_3_O_4_, FeO and metallic iron. Thus, this study provides a novel, economic and promising technology for treating the VOC pollutants.

## 1. Introduction

Volatile organic compounds (VOCs) are toxic pollutants, which are released in the environment through industrial emissions. As important contributors to particulate matter, surface ozone and secondary organic aerosols [1,2], their emissions are subjected to stringent legal restrictions [3,4]. Therefore, the development of new technologies to prevent the VOC contamination is currently of significant interest [5,6,7,8]. Among these, the processes that can enable the achievement of VOC resource utilization are a key to move towards sustainability; thus, these have received an increasing attention in recent years [9,10,11,12,13,14]. Considering that the mainstream technologies for VOCs elimination such as catalytic oxidation and thermal combustion are based on VOCs oxidation reactions, it is suspected that VOCs have a potential as reducing agents in the iron-making process from iron oxide ores.

Steel is the most commonly used material in today’s society, though its generation requires a significant amount of direct reduced iron (DRI) as the metal source. DRI is also known as sponge iron or pig iron, which has traditionally been produced through the reduction of iron ore using fossil fuels (coal and natural gas) as reducing agent and energy supply, thus, significantly contributing to the CO_2_ emissions in metallurgical industries [15,16]. As biomass is the most abundant, renewable and CO_2_ neutral resource, a number of research studies have explored the utilization of biomass and its derivatives (char or volatiles) for preparing DRI [17,18,19]. Bagatini et al. [20] demonstrated that biomass volatiles can serve as a viable reducing agent to reduce CO_2_ emissions in the industry. In fact, biomass volatiles mainly include non-condensable gases (CO, H_2_, CH_4_, C_2_H_6_, C_2_H_4_, C_2_H_2_, CO_2_, etc.) and liquid products (tar and oil like alkanes, aromatic hydrocarbons, alcohols, ethers, aldehydes, ketones, carboxylic acids, etc.), most of which belong to the VOC family in terms of their physiochemical properties [21,22]. Therefore, the reduction of iron oxides by VOCs can offer advantages for achieving multiple targets such as the purification and resource utilization of VOCs, acquisition of DRI co-product and carbon emission reduction during the iron-making process. For these reasons, the iron oxide oxidation-based approach differs from those already published in the literature in both process principle and material used. Among different types of iron oxides, hematite (Fe_2_O_3_) has the most abundant lattice oxygen. In this study, thus, a naturally hematite iron ore (HIO) was selected as the potential oxidizing agent to explore the feasibility of its reduction using *o*-xylene as a model VOC and investigate the effect of several process conditions on both *o*-xylene removal performance and hematite conversion. The results obtained in this study can provide a beneficial foundation for developing novel pathways for VOC resource utilization.

## 2. Results and Discussion

### 2.1. Reactivity of HIO with o-xylene

**Figure 1:**
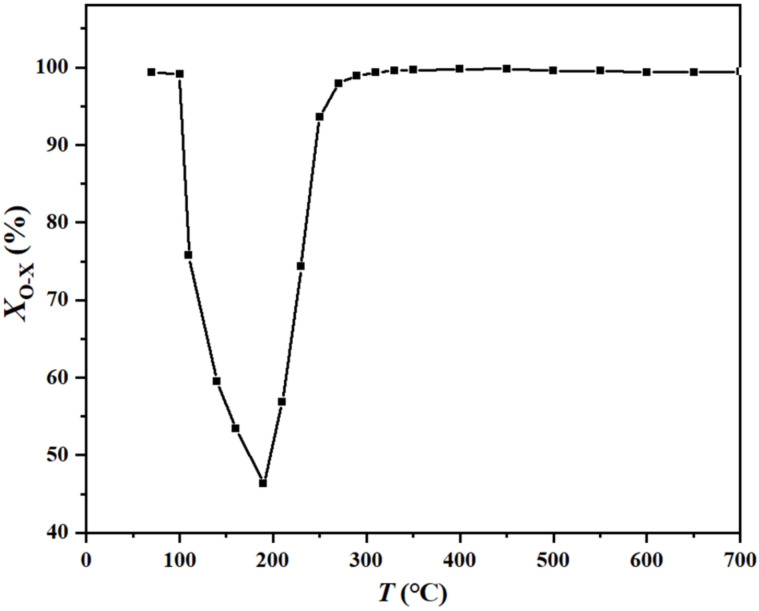
The plot of *o*-xylene removal ratios vs. temperature.

The chemical composition of HIO used in this study is presented in Table 1. The total iron content was 40.94%, whereas the content of Fe_2_O_3_ and iron lattice oxygen was calculated to be 58.53% and 17.59% respectively. As *o*-xylene passed through the heating HIO bed, Fe_2_O_3_ was reduced to iron species with low valence states (Fe_3_O_4_, FeO and Fe), while it was oxidized to CO_2_/CO and H_2_O, thus, achieving the dual goals of iron ore reduction and *o*-xylene removal. Figure 1 shows the plot of *o*-xylene removal ratios (*X_o_*_-x_) vs. temperature (*T*) for depicting the reactive removal profile of HIO for *o*-xylene as a function of temperature. The profile could be divided into three regions: an adsorption–desorption region (< 190 °C), an activation region (190~300 °C) and a reactive region (300~700 °C). In the adsorption–desorption region, a high *X_o_*_-x_ value was observed for *o*-xylene removal followed by a decline, which could be attributed to the physisorption process at low temperatures and thermal desorption at high temperatures. The amount of *o*-xylene removed by HIO increased with temperature in the activation region. Importantly, the reactive region covered a very wide temperature range (300~700 °C), and *o*-xylene was almost completely removed. It is important to note that, limited by the experimental conditions, the tests were performed before 700 °C. The results confirmed that HIO exhibited a quite high reactivity for *o*-xylene above 300 °C.

### 2.2. Effect of Temperature on o-xylene Removal and HIO Conversion

**Figure 2:**
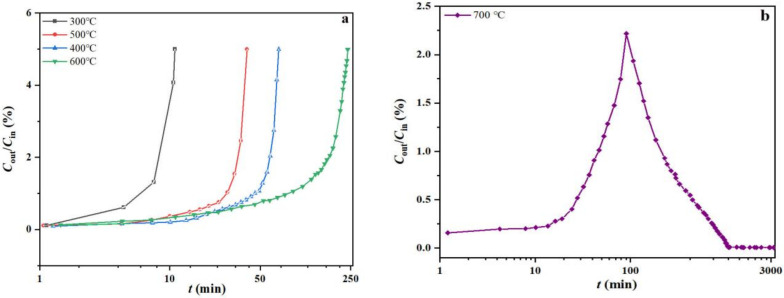
The reaction breakthrough curves of *o*-xylene at four different temperatures ranging 300–600 °C (**a**) and 700 °C (**b**).

To study the effect of temperature on *o*-xylene removal and HIO conversion, a group of reaction breakthrough curves were measured at 300, 400, 500, 600 and 700 °C (Figure 2a,b), in which the end point was set at *X_o_*_-x_ = 5%. Except for the experiment at 700 °C, each reaction breakthrough curve (Figure 2a) demonstrated an initial lag phase as well as a concave upward shape indicative of efficient removal ability, high filled bed utilization and process rate [23]. The calculated process parameters are listed in Table 2. As observed, *Q*_B_ exhibited a significant increase with temperature. Especially at 700 °C, the *o*-xylene breakthrough from the HIO bed remained unseen until 54 h, and the process curve was also different as compared to the curves acquired at lower temperatures (Figure 2b), thus, implying that HIO might have possessed super capacity (more than 691 mg g[Fe]^−1^) and unique reaction mechanism for removing *o*-xylene, which need to be studied further. Comparing *Q*_B_ of HIO for removing *o*-xylene with other materials already reported in the literature [9,10,11,12], the value obtained here was higher.

**Figure 3:**
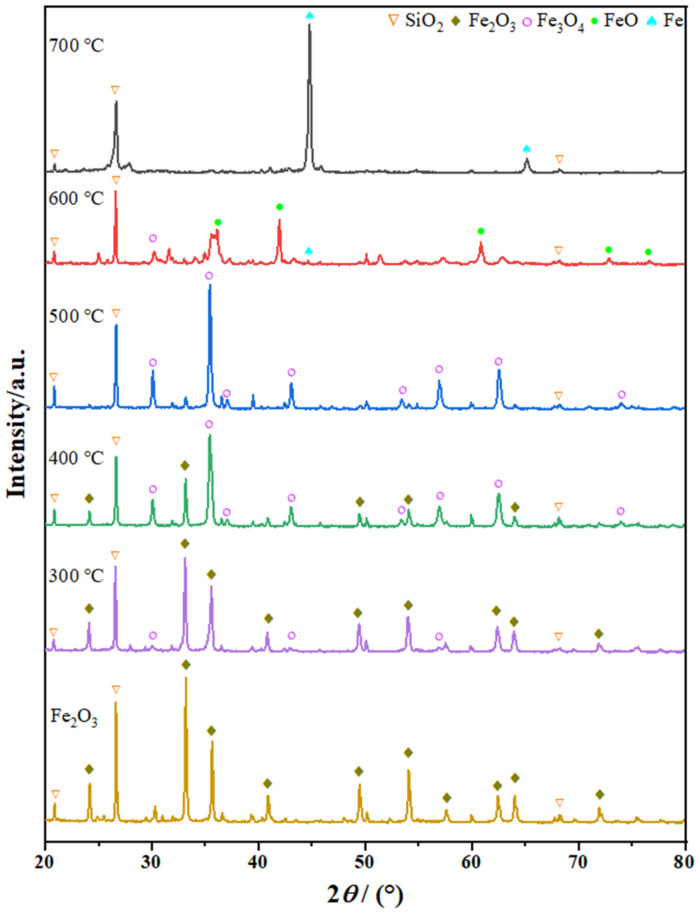
XRD patterns of HIO and its conversion products at different temperatures.

The obtained HIO mass loss due to the reduction by *o*-xylene, combined with TGA (Supplementary Material, Figure S1) and carbon data (Supplementary Material, Figure S2), allowed an estimation of the reduction degree (RD) at different temperatures. The estimated results are presented in Table 2, except for the experiment at 700 °C. In the temperature range 300–600 °C, RD was observed to increase with temperature, thus, exhibiting a similar behavior as *Q*_B_. At 300 °C, the results demonstrated only a minor reduction (2.79%), which was even lower than the theoretical RD value of Fe_2_O_3_ to Fe_3_O_4_ (11.1%). Thus, it was speculated that the sample derived from the reaction at 300 °C was composed of Fe_2_O_3_ and Fe_3_O_4_. At 400 and 500 °C, the RD values were observed to increase respectively to 14.05% and 15.73%, which were slightly higher than the theoretical RD value of Fe_2_O_3_ to Fe_3_O_4_ (11.1%). However, the values were significantly smaller than that of Fe_3_O_4_ to FeO (33.3%), thus, implying that the conversion product was predominantly Fe_3_O_4_ with a small amount of Fe_2_O_3_ or FeO. As the process was conducted at 600 °C, the RD value of HIO reached 36.68%, which was slightly higher than the theoretical RD value of Fe_2_O_3_ to FeO (33.3%), thereby suggesting that FeO was the main conversion product. Indeed, these notions were also confirmed by XRD analysis. Figure 3 presents the XRD patterns and phases of HIO and its conversion products at various temperatures. SiO_2_ (quartz, JCPDS 85-0798) was detected in all samples, however, with secondary importance in terms of intensity. SiO_2_ represents an inherent mineral impurity present in the pristine HIO and remains unchanged during the reactions with *o*-xylene. In terms of the iron species in the samples, HIO solely possessed Fe_2_O_3_ (hematite, syn, JCPDS 89-0597). On the other hand, the sample at 300 °C had Fe_2_O_3_ as the main phase but with a small amount of Fe_3_O_4_ (magnetite, JCPDS 99-0073). At 400 °C, both Fe_2_O_3_ and Fe_3_O_4_ became the main phases, whereas Fe_2_O_3_ turned into Fe_3_O_4_ as the temperature was raised to 500 °C. Further increasing the temperature (600 °C) led to the conversion of a majority of Fe_3_O_4_ into FeO (iron oxide, JCPDS 77-2355) and the formation of a small amount of metallic Fe (iron, JCPDS 99-0064). For the product obtained at 700 °C after 54 h (still remaining in the non-breakthrough state), both metallic Fe and quartz phases were detected in the XRD patterns. However, the EDS spectrum (Supplementary Material, Figure S2) of the product at 700 °C (using a mixture of 90% HIO and 10% pure carbon powder as the standard sample) revealed that it also possessed up to 10.8% carbon apart from Fe, O, Si, Al derived from the pristine HIO. As a comparison (Supplementary Material, Figure S2), only 0.34% carbon was observed in the EDS spectrum of the product at 600 °C, while no carbon deposition occurred at lower temperatures. The results demonstrated that the interaction of *o*-xylene with metallic Fe at 700 °C led to a significant level of carbon deposition, thus, resulting in the high *o*-xylene removal ability of hematite and getting a mixture of iron and carbon. Such a mixture is well known as a virgin source for high quality steel production, and its use can be effective in decreasing CO_2_ emissions in existing plants [16,24].

### 2.3. Effect of Flow Rate on o-xylene Removal and HIO Conversion

**Figure 4:**
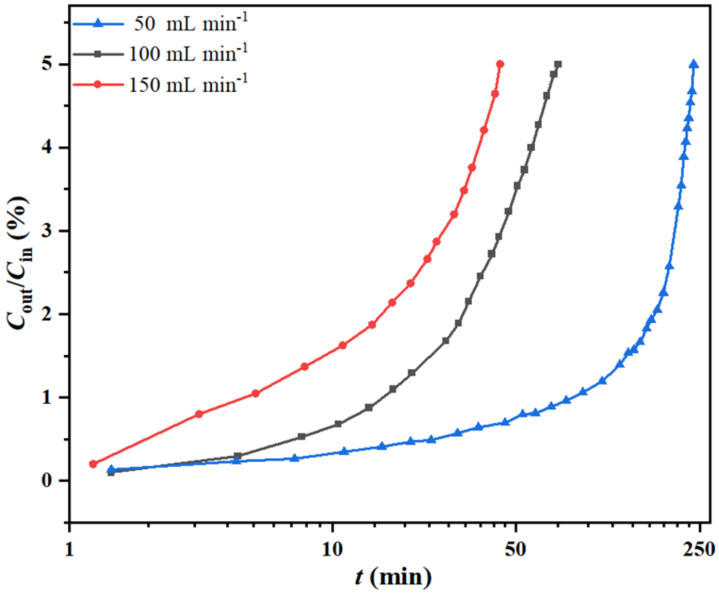
Reaction breakthrough curves of *o*-xylene at different flow rates.

The effect of flow rate (*V*_g_) on the *o*-xylene removal and degree of reduction in the HIO filled bed (bed length = 5.0 cm) was studied by varying the *V*_g_ value from 50 to 150 mL min^−1^ at 600 °C and a fixed *C*_in_ value of 7.0 mg L^−1^. The reaction breakthrough curves and corresponding process parameters are shown in Figure 4 and Table 3. The parameters *t*_B_, *Q*_B_ and RD were observed to decline on increasing *V*_g_. At a relatively low *V*_g_ value, HIO experienced a greater contact time with *o*-xylene in gaseous stream for carrying out the redox reaction. As the *V*_g_ value was increased, *o*-xylene molecules had a much shorter contact time for the redox reaction, which resulted in a poor degree of HIO reduction. Therefore, short *t*_B_ as well as low *Q*_B_ and RD were observed at the high flow rates.

### 2.4. Effect of Inlet Concentration of o-xylene on Its Removal and HIO Conversion

**Figure 5:**
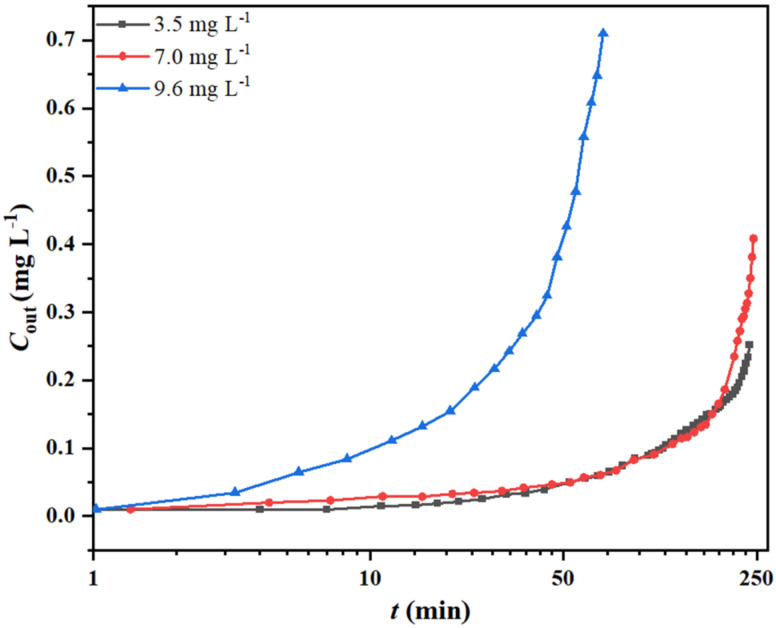
Reaction breakthrough curves of *o*-xylene at different inlet concentrations.

The effect of *C*_in_ (3.5, 7.0 and 9.6 mg L^−1^) on the *o*-xylene removal and degree of reduction in the HIO filled bed was analyzed at 600 °C, *V*_g_ of 50 mL min^−1^ and bed length of 5.0 cm. The results are shown in Figure 5, and the process parameters are also presented in Table 3. As observed, *t*_B_ at 3.5 mg L^−1^ *C*_in_ ≈ that of 7.0 mg L^−1^ >> that of 9.6 mg L^−1^. Both *Q*_B_ and RD were observed to reach their maximum values at 7.0 mg L^−1^. At a constant residence time, the rate of reaction of *o*-xylene with HIO increased with inlet concentration, thereby decreasing *t*_B_ [25]. However, the driving force and rate of the *o*-xylene oxidation were higher at higher *C*_in_ values, thus, facilitating the *o*-xylene elimination. Hence, the combination of these two factors provided the maximum *Q*_B_ and RD values (7.0 mg L^−1^).

## 3. Materials and Methods

### 3.1. Materials

*o*-xylene (analytical grade; Tianjin Kermel Chemical Reagent Co. Ltd., Tianjin, China) was used without further purification. HIO powder was purchased from Shanghai New Materials Co. Ltd., Shanghai, China. Its chemical composition was determined by XRF analysis. For this purpose, it was dried at 105 °C, followed by pressing into circular discs by using a tableting machine. Subsequently, it was ground in an agate mortar and sieved to a particle size between 250 and 830 μm. High purity nitrogen gas (99.999%; Shijiazhuang Xisanjiao Practical Gas Co. Ltd., Shijiazhuang, China) was used as a carrying and purging gas.

### 3.2. Experimental Setup and Procedure

**Figure 6:**
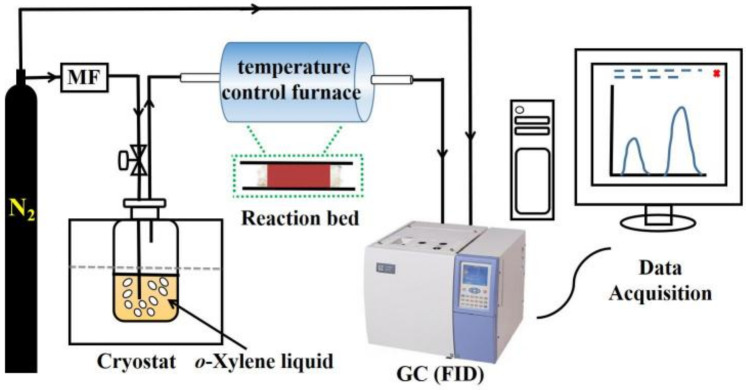
Schematic diagram of experimental setup.

The experiments to evaluate the *o*-xylene oxidation and HIO reduction were carried out in a horizontal tube furnace with an internal quartz tube of 10 mm diameter for heating the packed HIO particles, as shown in the schematic diagram in Figure 6. Prior to each test, 4.0 g HIO was packed in the central zone of the quartz tube and inserted in the furnace. The flow rate of *o*-xylene-containing N_2_ was controlled by using a flow meter (Sevenstar, D07-26, Beijing, China), and the concentration of *o*-xylene was monitored using a GC7900 gas chromatograph (Tianmei, Shanghai, China) equipped with a polyethylene glycol (PEG)-20M capillary column (30 m × 0.32 mm i.d.) and a flame ionization detector. To investigate the *o*-xylene removal performance by HIO and effects of primary process conditions, three separate experiments were performed:

(1) The reactivity was examined using a temperature-programmed mode from ambient temperature to 700 °C at a rate of 5 °C min^−1^. The gas flow rate (*V*_g_) of 50 mL min^−1^ and *o*-xylene inlet concentration (*C*_in_) of 7.0 mg L^−1^ were used. The results were presented in the form of a plot of *o*-xylene removal ratios (*X_o_*_-x_) vs. temperature (*T*); where *X_o_*_-x_ was calculated from Equation (1). From the experimental results, it was possible to identify the temperature range of the reactive region for the *o*-xylene-hematite system.

(2) The reaction breakthrough curves were obtained at a constant flow rate of 50 mL min^−1^ and an inlet concentration of 7.0 mg L^−1^ by varying the bed temperature in the temperature range of the reactive region to analyze its effect on the reaction. In this study, the breakthrough time (*t*_B_, min) was defined as the time at *C*_out, t_/*C*_in_ = 0.05, which was also set as an end point for each reaction breakthrough test. From this, the breakthrough removal capacity (*Q*_B_, mg g^−1^) was calculated by using Equation (2).

(3) Further reaction breakthrough tests of *o*-xylene were carried out by independently varying the flow rate or inlet concentration (all other variables were kept constant).

The reduction degree (RD) was determined for the obtained solid products, as calculated from Equation (3) [20,26].

(1) $$X_{O-X}=\frac{C_{\mathrm{in}}-C_{\mathrm{out},t}}{C_{\mathrm{in}}}\times 100\%$$

(2) $$Q_{B}=\frac{V_{g}C_{\mathrm{in}}}{m_{\mathrm{Fe}}}\int_{0}^{t_{B}}\left(1-\frac{C_{\mathrm{out},t}}{C_{in}}\right)dt$$

(3) $$\mathrm{RD}(\%)=[(m_{i}-m_{f})+m_{C}-W_{s}]\times 100/m_{\mathrm{Ored}}$$

where *m*_i_ and *m*_f_ are the initial and final mass of the sample (g), respectively; *m*_Fe_ is the mass of the iron element in sample; *t* is the adsorption time (min); *C*_out, *t*_ is the outlet *o*-xylene concentration in the gas flow (mg L^−1^); *m*_C_ and *W*_s_ are the carbon content and mass loss of the sample obtained by EDS and TGA analysis, respectively; and *W*_Ored_ is the mass of iron lattice oxygen in the raw sample. The other symbols such as *X_o_*_-x_, *Q*_B_, *t*_B_, *V*_g_, *C*_in_ and RD were defined earlier.

### 3.3. Characterization of Samples

The chemical composition of the HIO sample was determined by using X-ray fluorescence spectrometry (XRF; ARL PERFORM’X, Thermo Fisher Scientific, Waltham, MA, USA). The thermogravimetric analysis (TGA) was conducted using a TG/DSC system (PerkinElmer-STA8000 instrument (Fremont, CA, USA)) from 30 to 920 °C at a rate of 20 °C/min under N_2_ atmosphere with a gas flow of 20 mL/min. The particle morphology and composition analyses of the samples were performed using a cold-field emission scanning electron microscope (SEM; S-4800, Hitachi, Japan) equipped with an energy dispersive spectrometer (EDS; INCA 350, Hitachi, Japan) accessory. The X-ray diffraction spectra were obtained using a D8 Advance X-ray diffractometer (XRD; Brucker AXS, Karlsruhe, Germany).

## 4. Conclusions

The results presented in this study demonstrate the potential of using hematite ore as an oxidizer for simultaneously removing *o*-xylene gas and reducing iron oxides in the reactive region (300~700 °C). The removal capacity was found to be dependent on the temperature, flow rate and inlet concentration, while the conversion products of Fe_2_O_3_ mainly depended on the temperature. The increment in temperature from 300 °C to 700 °C led to a gradual reduction of Fe_2_O_3_ to Fe_3_O_4_, FeO and metallic iron, and the degree of reduction increased in turn. At 600 °C, the *Q*_B_ value reached 49.88 mg g[Fe]^−1^, while the value exceeded 691 mg g[Fe]^−1^ at the temperature up to 700 °C. The reduction products obtained from hematite, especially the mixture of metallic iron and carbon, are expected to be used as raw materials for the iron- and steel-making industries. Therefore, the findings observed in the current study offer a potential novel strategy for the purification and resource utilization of the VOC exhaust gas. Future efforts may further explore the applicability of the approach for more types of VOCs and its practical application.

## Figures and Tables

**Table 1:** Chemical composition of HIO (mass %).

Components	Fe	O	Si	Al	P	Ca	Sr	Mg	Mn	K	Ti	Others
Content	40.94	35.41	9.63	4.06	1.16	1.71	0.76	0.46	0.56	0.56	0.22	4.53

**Table 2:** Parameters for *o*-xylene removal and HIO conversion at different temperatures.

Parameters	Temperature for Obtaining Product (°C)				
	300	400	500	600	700
*t*_B_ (min)	11.00	39.62	69.65	237.32	3240^a^
*Q*_B_ (mg g[Fe]^−1^)	2.32	8.38	14.73	49.88	691^a^
RD (%)	2.79	14.05	15.73	36.68	--

^a^ artificial cutoff time in this study and its corresponding adsorption amount. --: not calculated due to the carbon deposition.

**Table 3:** Parameters for *o*-xylene removal and HIO conversion as a function of *V*_g_ and *C*_in_.

Parameters	*V*_g_ (mL min^−1^)			*C*_in_ (mg L^−1^)		
	50	100	150	3.5	7.0	9.6
*t*_B_ (min)	237.32	72.48	43.58	238.08	237.32	62.69
*Q*_B_ (mg g^−1^)	49.88	30.21	27.21	23.74	49.88	18.00
RD (%)	36.68	26.74	23.72	31.47	36.68	26.54

## Data Availability

Data is contained within the article or Supplementary Materials. The data presented in this study are available in Supplementary Materials.

## Supplementary Materials

The following supporting information can be downloaded online. Figure S1: TGA thermogram of HIO; Figure S2: SEM images and EDS spectra of the products at different temperatures.

## Sample Availability

Sample of the HIO. is available from the authors.
