# Supplementary material for: Utilization of Hematite Particles for Economical Removal of o-xylene in a High-Temperature Gas-Solid Reactor
Source: Molecules. 2022 Feb 23;27(5):1509. doi: 10.3390/molecules27051509 (PMC8911638; doi:10.3390/molecules27051509)
Supplement: Supplementary file 1 [file molecules-27-01509-s001.zip › molecules-1593506-supplementary.pdf]

Supplementary Material

Utilization of Hematite Particles for Economical Removal of *O*-xylene in a High-Temperature Gas-Solid Reactor

Xiaolong Ma [1], Dandan Zhao [2], Jinjin Qian [2], Zichuan Ma [2,*] and Jiansheng Cui [1,*]

Xiaolong Ma [1], Dandan Zhao [2], Jinjin Qian [2], Zichuan Ma [2,*] and Jiansheng Cui [1,*]

[1] School of Environmental Science and Engineering, Hebei University of Science and Technology, Shijiazhuang 050018, China; maxiaolong2410@hebust.edu.cn
[2] Hebei Key Laboratory of Inorganic Nano-materials, College of Chemistry and Material Sciences, Hebei Normal University, Shijiazhuang, 050024, China; zhaoddv@163.com (D.Z.); qianjinjin0209@163.com (J.Q.)
* Correspondence: mazc@hebtu.edu.cn (Z.M.); cuijiansheng@hebust.edu.cn (J.C.); Tel: +86 0311 80787400 (Z.M.)

Contents

Figure S1. TGA thermogram of HIO…………...3
Figure S2. SEM images and EDS spectra of the products at different temperatures…….........................3

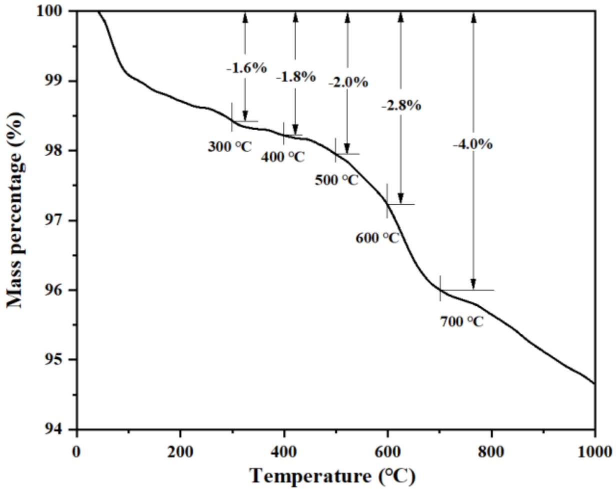

Figure S1. TGA thermogram of HIO.

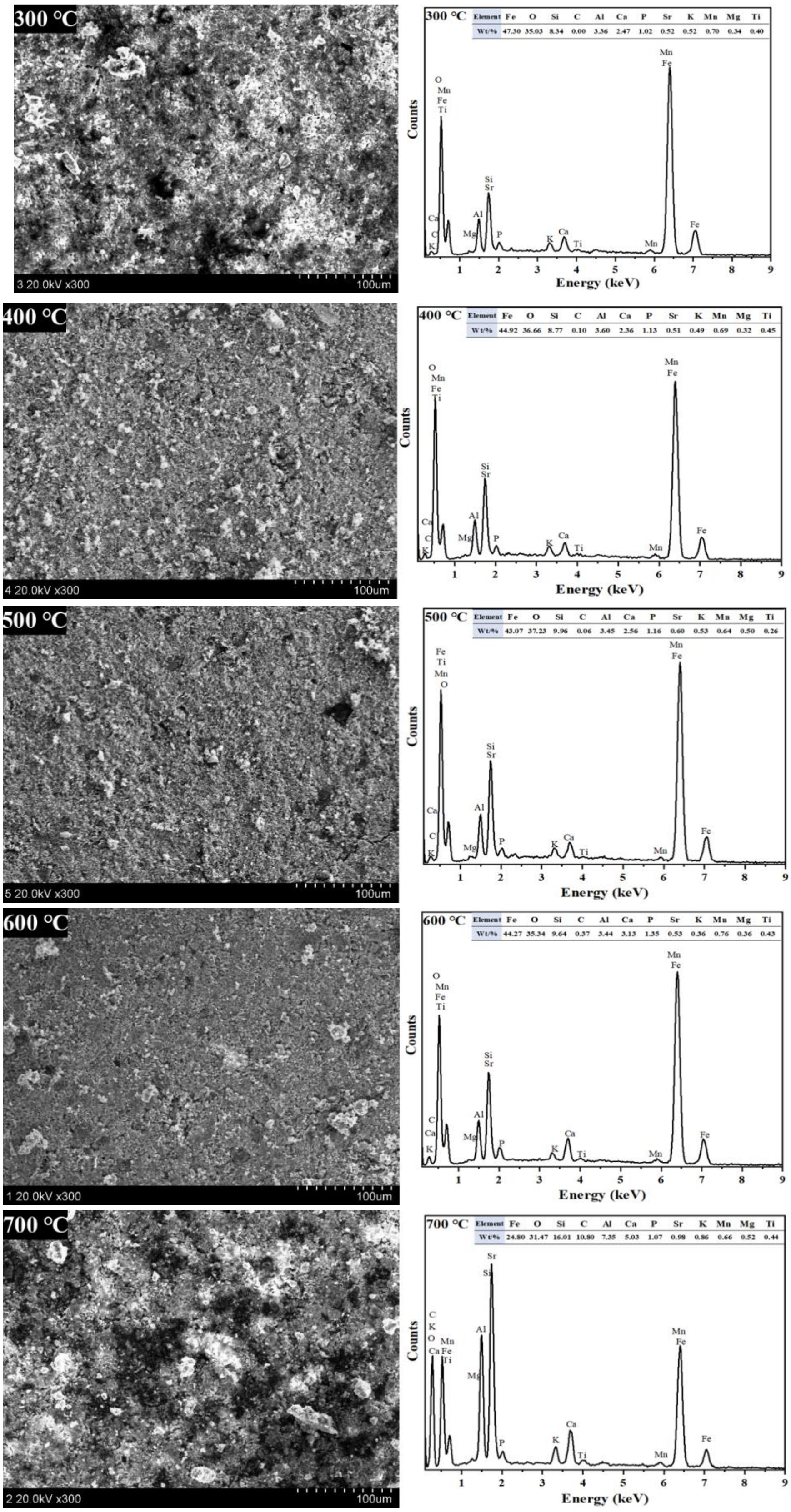

Figure S2. SEM images and EDS spectra of the products at different temperatures.
